# Genome-wide association study identifies zonisamide responsive gene in Parkinson’s disease patients

**DOI:** 10.1038/s10038-020-0760-8

**Published:** 2020-05-01

**Authors:** Pei-Chieng Cha, Wataru Satake, Yuko Ando-Kanagawa, Ken Yamamoto, Miho Murata, Tatsushi Toda

**Affiliations:** 1 Division of Neurology/Molecular Brain Science, Kobe University Graduate School of Medicine, Hyogo, Japan; 2 Department of Genomic Medicine, National Cerebral and Cardiovascular Center, Osaka, Japan; 3 Department of Neurology, Graduate School of Medicine, The University of Tokyo, Tokyo, Japan; 4 Department of Medical Biochemistry, Kurume University School of Medicine, Fukuoka, Japan; 5 Department of Neurology, National Center Hospital, National Center of Neurology and Psychiatry, Tokyo, Japan

**Keywords:** Genome-wide association studies, Genetics research

## Abstract

Long-term treatment of Parkinson’s disease (PD) by levodopa leads to motor complication “wearing-off”. Zonisamide is a nondopaminergic antiparkinsonian drug that can improve “wearing-off” although response to the treatment varies between individuals. To clarify the genetic basis of zonisamide responsiveness, we conducted a genome-wide association study (GWAS) on 200 PD patients from a placebo-controlled clinical trial, including 67 responders whose “off” time decreased ≥1.5 h after 12 weeks of zonisamide treatment and 133 poor responders. We genotyped and evaluated the association between 611,492 single nucleotide polymorphisms (SNPs) and “off” time reduction. We also performed whole-genome imputation, gene- and pathway-based analyses of GWAS data. For promising SNPs, we examined single-tissue expression quantitative trait loci (eQTL) data in the GTEx database. SNP rs16854023 (Mouse double minute 4, *MDM4*) showed genome-wide significant association with reduced “off” time (*P*_Adjusted_ = 4.85 × 10^−9^). Carriers of responsive genotype showed >7-fold decrease in mean “off” time compared to noncarriers (1.42 h vs 0.19 h; *P* = 2.71 × 10^−7^). In silico eQTL data indicated that zonisamide sensitivity is associated with higher MDM4 expression. Among the 37 pathways significantly influencing “off” time, calcium and glutamate signaling have also been associated with anti-epileptic effect of zonisamide. *MDM4* encodes a negative regulator of p53. The association between improved motor fluctuation and MDM4 upregulation implies that p53 inhibition may prevent dopaminergic neuron loss and consequent motor symptoms. This is the first genome-wide pharmacogenetics study on antiparkinsonian drug. The findings provide a basis for improved management of “wearing-off” in PD by genotype-guided zonisamide treatment.

## Introduction

Parkinson’s disease (PD) is a chronic progressive movement disorder in which loss of dopaminergic neurons in the *substantia nigra* of the midbrain leads to motor symptoms including tremor, bradykinesia, rigidity, and postural instability [1]. Previous studies suggest that the neurodegeneration results from the interplay of intrinsic as well as extrinsic factors including genetic mutations, altered activity patterns of ion channels such as l-type calcium channels and ATP-sensitive potassium channels, reduced neurotransmission of dopaminergic neurons, lysosomal and mitochondrial dysfunction, α-synuclein accumulation, and neurotoxic stress [2].

Current clinical management of PD is largely confined to symptomatic treatment, with levodopa being the most effective drug. Conversion of levodopa to dopamine compensates the loss of dopamine resulting from degeneration of dopaminergic neurons in the *substantia nigra* of PD patients. However, due to the short half-life of levodopa and the continuous loss of dopaminergic neurons following disease progression, beneficial effect of each dose of the drug gets shorter. As a result, motor and nonmotor symptoms re-emerge in PD patients before the next dose of levodopa is being taken. This phenomenon, which termed “wearing-off”, normally appears after several years of levodopa treatment and can significantly affect quality of life of PD patients [3]. “Wearing-off” is often managed by altering the dosing, timing, and formulation of levodopa to prolong the effect of the drug. Co-administration of other drugs such as monoamine oxidase (MAO)-B inhibitors, catechol-O-methyl transferase inhibitors, and dopamine agonists that have longer half-life has also been shown to be useful for extending the effect of levodopa [4].

Zonisamide is a sulfonamide anti-epileptic drug that improves “wearing-off” in PD patients without increasing dyskinesia [5]. In Japan, zonisamide was approved as an adjunctive treatment for PD in 2009. The efficacy of zonisamide in improving motor symptoms and reducing “off” time in PD patients has been evaluated in a series of double-blind placebo-controlled clinical trials [5–7]. Owing to good safety profiles and limited interactions with other drugs, zonisamide has been used as an anti-convulsant since 1989 and has demonstrated beneficial effects in various neurological and psychiatric diseases [8].

Previous studies suggest that zonisamide may exert its anti-epileptic and antiparkinsonian effects through inhibition of sodium and T-type calcium channels, and MAO-B activity, as well as regulation of striatal delta1-receptor-associated gamma aminobutyric acid (GABA)ergic neurotransmission [9–11]. More recently, an investigation of the antiparkinsonian effect of zonisamide revealed a potential role against oxidative stress and dopaminergic neurodegeneration [12, 13].

Although inter-individual variation in the response to zonisamide treatment has been documented in clinical settings [14], the genetic basis for this observation has yet to be explored. We have previously identified common variants associated with PD through a genome-wide association study (GWAS) [15]. Here, we report a GWAS that investigates the association between genetic variations and response to zonisamide treatment in Japanese PD patients.

## Subjects/materials and methods

### Subjects

Subjects were Japanese PD patients who took part in a multicenter, randomized, double-blind, placebo-controlled study that aimed to determine the efficacy of zonisamide in treating “wearing-off” [7]. The subjects of the clinical trial have mean daily “off” time of at least 2 h for the last 7 days of the run-in period. These patients have been treated with any combination drugs of levodopa and dopa decarboxylase inhibitor for at least 6 consecutive months and had responded to levodopa during the first few years of levodopa treatment. Among the 373 clinical trial participants whose DNA samples were available for the current study, 119 who received a placebo were excluded along with 32 subjects who lacked a record on dosing information, one who lacked UPDRS part III total score or “off” time values at week 12, and 19 who did not complete the clinical trial either due to adverse events, withdrawal from the study, investigator’s discretion, or worsening of PD. The GWAS was conducted for 202 subjects who received either 25 or 50 mg zonisamide per day and who had a complete record of changes in “off” time from baseline after 12 weeks of treatment. A flowchart detailing the selection of samples is shown as Supplementary Fig. 1. All participants have provided written informed consent to participate in the current pharmacogenetic study when provided informed consent to take part in the clinical trial in accordance with the process approved by Ethical Committee at each of the medical institution where the patients were enrolled.

### Definition of zonisamide responsiveness

Responsiveness to zonisamide was evaluated as efficacy of the drug in reducing “off” time of PD patients. Patients who showed a decrease of at least 1.5 h from baseline in “off” time after 12 weeks of zonisamide treatment were defined as responders; whereas all the remaining subjects were classified as poor responders. Briefly, all subjects were instructed to record in a diary their “on” and “off” states as well as the occurrence of dyskinesia for every 30 min that they were awake. The “on” stage was defined as the effective time of the medication when symptoms were well controlled, whereas the “off” stage referred to the stage when the medication was no longer effective, and symptoms re-emerged. “Off” time was calculated based on information in the diary, considering the last 7 days before each hospital visit (except for the screening visit). When “off” time data for fewer than 5 days were available, the data obtained at the visit were handled as missing. Further details regarding clinical assessment of patients are described in the paper detailing the clinical trial [7]. Demographic and clinical characteristics of subjects included in the final analysis are summarized in Table 1.

**Table 1:** Characteristics of responders and poor responders defined on change in “off” time from baseline

Variables	Responsiveness defined based on change in “off” time from baseline		
	Responders, R (*n* = 67)	Poor responders, PR (*n* = 133)	*P* value (R vs PR)*
Female sex (%)	41/67 (61.2%)	78/133 (58.6%)	
Age (Year, Mean ± SD)	64.6 (7.2)	63.2 (7.6)	0.5073
BMI (Mean ± SD)	22.6 (3.8)	22.7 (3.7)	0.5527
Onset age of PD (Year, Mean ± SD)	56.6 (9.0)	54.8 (8.3)	0.3886
Duration from onset of PD (Year, Mean ± SD)	8.0 (4.7)	8.4 (4.3)	0.5809
Onset age of “wearing-off” (Year, Mean ± SD)	62.2 (8.3)	60.6 (8.2)	0.3520
Duration from onset of “wearing-off” (Year, Mean ± SD)	2.4 (3.0)	2.6 (3.0)	0.4675
MMSE (Mean ± SD)	28.3 (2.0)	28.5 (2.0)	0.8391
Modified Hoehn & Yahr score (ON) (Mean ± SD)	2.2 (0.8)	2.3 (0.7)	0.9305
Modified Hoehn & Yahr score (OFF) (Mean ± SD)	3.2 (0.8)	3.4 (0.8)	0.2974
Dose of levodopa (mg/day, Mean ± SD)	1781.7 (639.0)	1907.9 (708.3)	0.0819
LEDD^a^ (mg/day, Mean ± SD)	469.9 (164.3)	498.0 (170.9)	0.1114
Number of concomitant drugs (Mean ± SD)	3.0 (1.1)	3.3 (1.1)	0.7516
UPDRS part III total score at week 12 (Mean ± SD)	13.1 (9.6)	16.0 (10.8)	0.5177
UPDRS part III total score at baseline (Mean ± SD)	16.8 (10.3)	19.1 (11.4)	0.9439
Change in UPDRS part III total score from baseline (Mean ± SD)	−3.8 (5.8)	−3.1 (6.5)	0.2230
Average “Off” time at week 12^b^ (Hour, Mean ± SD)	3.8 (2.3)	6.7 (2.6)	0.0010
Average “Off” time at baseline^b^ (Hour, Mean ± SD)	6.5 (2.4)	6.2 (2.1)	0.7917
Change in “off” time from baseline (Hour, Mean ± SD)	−2.8 (1.3)	0.5 (1.5)	5.95 × 10^−7^

*SD* standrad deviation, *BMI* body mass index, *PD* Parkinson’s disease, *MMSE* Mini–Mental State Examination, *LEDD* levodopa-equivalent daily dose

*P values were determined by using two-tailed unpaired t-test with equal variance

^a^Conversion factor for LEDD: bromocriptine mesilate, ×10; cabergoline, ×70; pergolide mesilate, ×100; pramipexole hydrochloride hydrate, ×60

ropinirole hydrochloride, ×16.67; talipexole hydrochloride, ×60

^b“^Off” time was calculated using patients’ diary information for the last 7 days before each visit (excluding screening visit)

### Genotyping and quality controls (QCs)

GWAS was performed by using Illumina Infinium HumanOmniExpressExome-8_v1.2 BeadChip (Illumina, San Diego, CA, USA). The 202 PD patients were genotyped for 964,193 SNPs (of which 273,246 were exonic markers). As QC, 11 subjects were genotyped twice to assess concordance of genotyping. SNPs with a call rate <99%, deviating from the Hardy–Weinberg equilibrium (*P* ≤ 1 × 10^−6^), and those that were monomorphic or nonautosomal were excluded from the analysis. Population stratification was evaluated by principal component analysis (PCA) using EIGENSOFT v.5.0.2 software [16], with the four HapMap [17] populations—namely, Europeans, Africans, and East Asians (Japanese and Han Chinese)—serving as reference groups. Degree of relatedness between samples was examined with the “--genome” function of PLINK v.1.07 [18].

### Statistical analysis

Associations between SNPs and responsiveness to zonisamide were assessed with the Cochran–Armitage trend test and multivariate logistic regression analysis (PLINK v.1.07). For logistic regression analysis, daily dose of zonisamide administered by patients and baseline “off” time of patients were included as additional covariates to predict zonisamide responsiveness. Three genetic models, namely additive, dominant, and recessive models, were examined in multivariate logistic regression analysis, with the minor allele found in the responders was considered as reference allele. Model producing the smallest *P* value was used to determine the mode of inheritance of the SNPs. The significance threshold for the GWAS was set at 5 × 10^−8^. A Manhattan plot was generated using Haploview v.4.1 [19] based on the *P* value from the trend test. Genomic inflation factor (λgc) was determined and a quantile–quantile (QQ) plot was generated using R statistical software v.3.13 (http://www.R-project.org/). A regional association plot generated using LocusZoom [20]. Box and whisker plots (R statistical software v.3.13) were used to graphically illustrate the association between clinical variables (including changes in “off” time) and SNP genotypes. The Kruskal–Wallis and Mann–Whitney *U* tests (R statistical software v.3.13) were used to assess differences in the distribution of clinical variables based on SNP genotypes. Single-tissue eQTL data of SNP were retrieved from the GTEx portal (https://www.gtexportal.org).

### Whole-genome imputation (WGI)

WGI was conducted with data for 286 East Asians in 1000 Genome [21] (Phase Iv3 2010-11, data freeze on 20120314). Markers with minor allele frequencies <1% were excluded from the WGI analysis, which was performed using minimac2 [22] the subsequent association analysis was performed using mach2dat (http://www.unc.edu/~yunmli/software.html) with the daily dose of zonisamide included as a covariate. Markers with a quality score of *R*^2^ < 0.9 were excluded from the association analysis. Genome-wide significance thresholds were set at *P* = 8.61 × 10^−9^ for WGI analysis considering the markers investigated.

### Gene-based association analysis

Gene-based association analysis was performed using Versatile Gene-based Association Study 2 [23]. Briefly, after assigning SNPs to genes based on hg19 genomic coordinates, gene-based *P* values were calculated using a simulation method that considers linkage disequilibrium (LD) between markers in a gene. Data from the Asian population in the 1000 Genomes Project were used for LD estimation. Gene boundary was defined as ±50 kb for SNP selection, and only the top 10% of associated SNPs within each gene were included in the analysis. Genome-wide significance thresholds were set at 2.11 × 10^−6^ for gene-based association analysis considering 23,735 genes investigated.

### Pathway-based analysis

Pathway-based analysis was carried out using Gene Set Analysis-SNP [24]. Briefly, SNPs were assigned to the gene if they were located within ±20 kb of the gene locus. In the case where more than one SNP was mapped to a gene, the *P*-value of the gene was represented by the second most significant *P* value of all SNPs mapped to the gene to avoid false positive association arising from random associations as discussed in Nam et al. [24]. Pathways analyzed in this study were downloaded from the Molecular Signatures Database v.5.0 [25]. The analyzed pathways include 1330 gene sets in the canonical pathways (Biocarta, Kyoto Encyclopedia of Genes and Genomes, and Reactome) and 1434 gene ontology (GO) terms (including biological processes, cellular components, and molecular functions). The significance of the association between gene sets and reduction in “off” time was determined using the Bonferroni-corrected *P* value. Genome-wide significance thresholds were set at 3.76 × 10^−5^ and 3.44 × 10^−5^ for canonical pathways and GO terms, respectively.

### Association between SNPs and improvement in motor symptoms measured by Unified PD Rating Scale (UPDRS) Part III total score

Responsiveness to zonisamide was measured as a reduction in UPDRS Part III total score, which measures motor symptoms of PD patients. Patients who showed a decrease in UPDRS Part III total score of at least 5 points from baseline after 12 weeks of zonisamide treatment were defined as responders; and all remaining subjects were considered as poor responders. SNP genotyping and SNP-, gene-, and pathway-based association analyses were performed as described above.

## Results

### Quality controls (QCs) and GWAS

The overall genotyping rate for subjects and genotyping consistency for all duplicates exceeded 99.8%. PCA with HapMap populations as reference groups confirmed that all subjects were of East Asian origin. A subsequent PCA analysis limited to the subjects of the present study revealed two outliers (Supplementary Fig. 2a, b) that were excluded from subsequent analyses. All subject pairs showed a low degree of relatedness, with a PI_HAT value <0.05. A genomic inflation factor (λgc) of 1.068 in the QQ plot (Supplementary Fig. 3) indicated negligible population sub-stratification among subjects. After QC, we identified 611,492 SNPs in 200 PD patients who received zonisamide at 25 or 50 mg/day (*n* = 100 each) and were available for the analysis (Supplementary Fig. 1).

Among the 200 PD patients analyzed, 67 who responded very well to zonisamide by showing a decrease of at least 1.5 h in “off” time from baseline after 12 weeks of treatment were designated as responders; whereas the remaining 133 subjects were classified as poor responders. As summarized in Table 1, responders achieved a mean decrease in “off” time of 2.8 h; whereas poor-responders showed an average increase in “off” time of 0.5 h after 12 weeks of zonisamide treatment. Responders and poor responders were matched in terms of demographic and clinical characteristics. Importantly, the two groups exhibited no obvious difference in baseline “off” time value or onset age and duration from onset of “wearing-off”.

Based on the Manhattan plot of the GWAS data, SNP rs16854023 (chromosome 1q32.1) showed a genome-wide significant association (*P*_Trend_ = 4.23 × 10^−8^) with a reduction in “off” time (Supplementary Fig. 4). A strong association between this SNP and a reduction in “off” time was observed in patients administered zonisamide at 25 mg/day (*P* = 2.44 × 10^−4^) and 50 mg/day (*P* = 3.00 × 10^−5^) (Table 2). This was confirmed by a logistic regression analysis in which daily dose of zonisamide and baseline “off” time were covariates (*P*_Adjusted_ = 4.85 × 10^−9^). Allele C is detected as minor allele in responders and is associated with lower drug responsiveness (OR = 0.133). The GWAS identified 13 markers that showed suggestive associations (*P*_Trend_ < 10^−5^) with the responsiveness to zonisamide treatment (Supplementary Table 1). A further association analysis after accounting for the effects of the marker SNP did not reveal any additional associations that achieved genome-wide significance.

**Table 2:** SNP showing genome-wide significant association with reduction in “off” time in PD patients administered zonisamide

Chr.	SNP	Location	A1/A2	Zonisamide	Responders				Poor-responders				*P*-value*	Logistic regression analysis^a^			Nearest	Location
				dose (mg/day)	CC	CT	TT	MAF	CC	CT	TT	MAF		*P*min	OR	Model	gene	
1	rs16854023	204551830	C/T	50	4	11	22	0.26	15	40	8	0.56	3.00 × 10^−5^				*MDM4*	Intron
				25	2	9	19	0.22	16	38	16	0.50	2.44 × 10^−4^					
				All	6	20	41	0.24	31	78	24	0.53	4.23 × 10^−8^	4.85 × 10^−9^	0.133	DOM		

*Chr.* Chromosome, *SNP* single nucleotide polymorphism, *A1* Allele 1 (minor allele detected in Responders); A2 Allele 2 (major allele detectedin Responders), *MAF* minor allele frequency; Pmin Minimum *P* value, OR odds ratio, DOM dominant model

*P value of Armitage Trend test

^a^Logistic regression analysis considering daily dose of zonisamide as well as baseline “off” time value as covariates; three models of inheritance, namely allelic,dominant, and recessive were considered. Genetic model that gave the minimum *P* value was shown

### Whole-genome imputation (WGI)

**Fig. 1:**
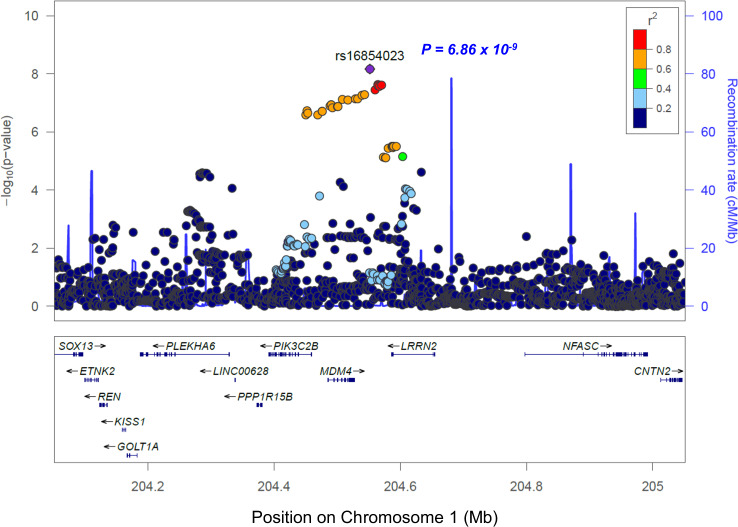
Regional plot for the most associated region on Chromosome 1q32.1. Regional plot was generated based on the association data from whole-genome imputation analysis by using 1000 Genome data of the 286 East Asians. cM centiMorgan, Mb Megabases

To increase the study resolution, we performed WGI to infer the genotypes of SNPs that were not investigated in the GWAS. Associations between changes from baseline in “off” time after 12 weeks of treatment and the 5,805,599 markers with imputation quality score of *R*^2^ ≥ 0.9 were examined. A total of 42 markers with *P* < 10^−6^ were identified (Supplementary Table 2). Two markers (including an SNP) located on chromosome 1q32 achieved a genome-wide significance threshold of *P* = 8.61 × 10^−9^. A regional plot (Fig. 1) based on WGI data indicated that the strongly associated region encompassed two genes, namely *mouse double minute 4 (MDM4)* and *leucine-rich repeat transmembrane neuronal 2 (LRRN2)*.

### Correspondence between changes in “off” time, genotypes of SNPs and eQTL data

**Fig. 2:**
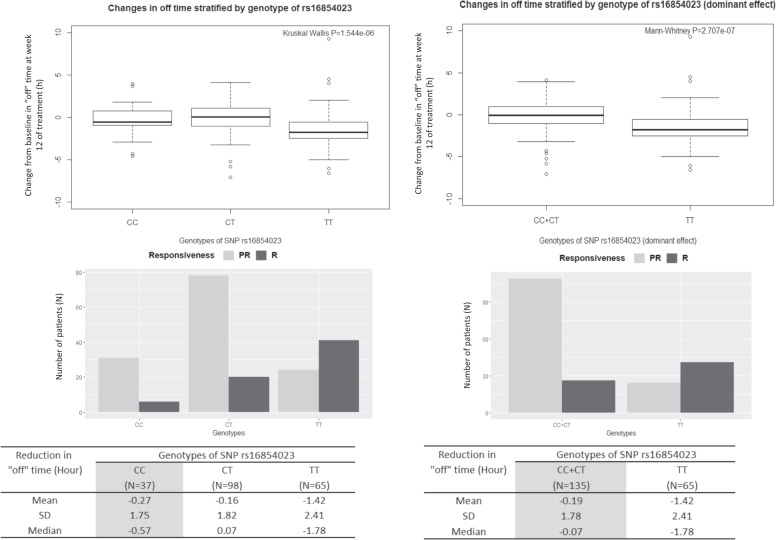
Distribution of “changes in “off” time from baseline at week 12” stratified by the genotypes of SNP rs16854023 as demonstrated by the Box and Whisker plot, histogram and table. *N* Number of subjects; PR poor responders; R responders; SD standard deviation; SNP single nucleotide polymorphism

We generated box and whisker plots to visualize the correspondence between changes in “off” time and genotypes of the marker SNP rs16854023 (Fig. 2). More than 60% of TT carriers were responders to zonisamide. On average, TT carriers showed a 1.42 h decrease in “off” time as compared to 0.19 h in carriers of the CT and CC genotypes. To determine the reason for the increased responsiveness to the drug among TT carriers, we used the GTEx portal to examine eQTL data for the SNP. Although the marker SNP has been reported to influence the expression of the phosphatidylinositol-4-phosphate 3-kinase catalytic subunit type 2β gene in esophageal mucosa (Supplementary Fig. 5), expression data in neurons and nerve tissue are lacking. We therefore investigated other SNPs on 1q32.1 that showed a suggestive association (*P*_Trend_ < 10^−5^) with reduction in “off” time in the GWAS (Supplementary Table 1) for which eQTL data in neuron and nerve tissue were available. Two SNPs (rs10900597 and rs2290854) with GWAS *P*_Trend_ = 7.62 × 10^−6^ that were in complete LD (*R*^2^ = 1) but had an *R*^2^ = 0.24 with the marker SNP (Supplementary Fig. 6) influenced MDM4 expression in tibial nerve (Supplementary Fig. 7a, b). A box and whisker plot illustrating changes in “off” time based on the genotypes of SNP rs10900597 (Supplementary Fig. 8) indicated that carriers of the CC responsive genotype showed an average decrease in “off” time of 2.19 h as compared to 0.09 h for carriers of the homozygous TT genotype. The associations between SNP (rs16854023 and rs10900597) genotypes and other clinical variables such as changes in UPDRS Part III total score after 12 weeks of treatment, plasma concentration of zonisamide at week 4 of treatment, age of PD/“wearing-off” onset, and duration from onset of PD/“wearing-off” were nonsignificant (Supplementary Fig. 9a, b).

### Gene- and pathway-based analysis

To further increase the coverage of our study by detecting combined effects of weaker signals from GWAS that may have been overlooked in conventional SNP-based GWAS, we carried out gene- and pathway-based association analyses. The former examined the associations between 23,735 genes and responsiveness to zonisamide. A Manhattan plot for gene-based GWAS (Supplementary Fig. 10) indicated that only the *MDM4* gene (*P*_gene_ = 2.0 × 10^−6^) was significantly associated with a decrease in “off” time.

For pathway-based analysis, 16 of the 1330 canonical pathways examined showed significant associations with a reduction in “off” time (*P*_Bonferroni-corrected_ < 3.76 × 10^−5^). On the other hand, of the 1454 GO terms examined, 21 showed a significant association (*P*_Bonferroni-corrected_ < 3.44 × 10^−5^) (Table 3). These pathways included calcium signaling pathway, potassium channels, transmembrane receptor protein phosphatase activity, extracellular matrix-related pathways, and Ras GTPase binding, among others.

**Table 3:** Pathways showing significant association with a reduction in “off” time after Bonferroni correction

MsigDB category	Pathways		Gene count	Set size	z-score	*P* value
**C2 canonical pathways**	**KEGG_CALCIUM_SIGNALING_PATHWAY**		**166**	**178**	**5.964**	**1.23** × **10**^−**9**^
	REACTOME_TRANSMISSION_ACROSS_CHEMICAL_SYNAPSES		174	186	5.816	3.01 × 10^−9^
	KEGG_ABC_TRANSPORTERS		42	44	5.015	2.66 × 10^−7^
	NABA_COLLAGENS		41	44	4.774	9.02 × 10^−7^
	REACTOME_ABC_FAMILY_PROTEINS_MEDIATED_TRANSPORT		32	34	4.734	1.10 × 10^−6^
	NABA_BASEMENT_MEMBRANES		38	40	4.685	1.40 × 10^−6^
	REACTOME_TRANSPORT_OF_INORGANIC_CATIONS_ANIONS_AND_AMINO_ACIDS_OLIGOPEPTIDES		88	94	4.591	2.20 × 10^−6^
	PID_REELIN_PATHWAY		29	29	4.463	4.05 × 10^−6^
	KEGG_ECM_RECEPTOR_INTERACTION		83	84	4.439	4.52 × 10^−6^
	**REACTOME_TANDEM_PORE_DOMAIN_POTASSIUM_CHANNELS**		**12**	**12**	**4.395**	**5.53** × **10**^−**6**^
	REACTOME_EFFECTS_OF_PIP2_HYDROLYSIS		22	25	4.304	8.38 × 10^−6^
	REACTOME_NEUROTRANSMITTER_RECEPTOR_BINDING_AND_DOWNSTREAM_TRANSMISSION_IN_THE_POSTSYNAPTIC_CELL		127	137	4.135	1.78 × 10^−5^
	NABA_ECM_GLYCOPROTEINS		182	196	4.134	1.79 × 10^−5^
	REACTOME_SIGNALING_BY_RHO_GTPASES		100	113	4.094	2.12 × 10^−5^
	KEGG_LONG_TERM_POTENTIATION		65	70	4.039	2.68 × 10^−5^
	REACTOME_PHOSPHOLIPID_METABOLISM		184	198	3.957	3.79 × 10^−5^
**C5 GO terms**	TRANSMEMBRANE_RECEPTOR_PROTEIN_PHOSPHATASE_ACTIVITY		19	19	5.837	2.65 × 10^−9^
	COLLAGEN		22	23	4.932	4.08 × 10^−7^
	METAL_ION_TRANSMEMBRANE_TRANSPORTER_ACTIVITY		138	147	4.754	9.97 × 10^−7^
	VOLTAGE_GATED_CHANNEL_ACTIVITY		69	73	4.730	1.12 × 10^−6^
	**GLUTAMATE_RECEPTOR_ACTIVITY**		**20**	**20**	**4.714**	**1.21** × **10**^−**6**^
	CELL_RECOGNITION		18	19	4.680	1.43 × 10^−6^
	**GLUTAMATE_SIGNALING_PATHWAY**		**16**	**17**	**4.678**	**1.45** × **10**^−**6**^
	EMBRYONIC_MORPHOGENESIS		17	17	4.627	1.86 × 10^−6^
	**VOLTAGE_GATED_CALCIUM_CHANNEL_ACTIVITY**		**17**	**18**	**4.615**	**1.97** × **10**^−**6**^
	ACTIVE_TRANSMEMBRANE_TRANSPORTER_ACTIVITY		114	122	4.542	2.78 × 10^−6^
	VOLTAGE_GATED_CATION_CHANNEL_ACTIVITY		63	66	4.509	3.26 × 10^−6^
	HYDROLASE_ACTIVITY_ACTING_ON_ACID_ANHYDRIDESCATALYZING_TRANSMEMBRANE_MOVEMENT_OF_SUBSTANCES		35	39	4.328	7.52 × 10^−6^
	ATPASE_ACTIVITY_COUPLED_TO_MOVEMENT_OF_SUBSTANCES		36	40	4.303	8.41 × 10^−6^
	EXTRACELLULAR_MATRIX_PART		53	57	4.302	8.45 × 10^−6^
	EXTRACELLULAR_MATRIX		95	100	4.296	8.70 × 10−^6^
	RAS_GTPASE_BINDING		25	25	4.222	1.21 × 10^−5^
	PROTEINACEOUS_EXTRACELLULAR_MATRIX		93	98	4.142	1.72 × 10^−5^
	SMALL_GTPASE_BINDING		32	33	4.087	2.19 × 10^−5^
	PRIMARY_ACTIVE_TRANSMEMBRANE_TRANSPORTER_ACTIVITY		36	40	4.034	2.74 × 10^−5^
	GTPASE_BINDING		33	34	4.022	2.88 × 10^−5^
	CATION_CHANNEL_ACTIVITY		113	119	3.990	3.31 × 10^−5^

Pathways written in bold are those have been previously reported to be associated with mechanisms of action of zonisamide

### Association between SNPs and improvement in motor symptoms measured by Unified Parkinson’s Disease Rating Scale (UPDRS) Part III total score

Given that zonisamide has been reported to improve motor symptoms of PD patients [6, 26] a GWAS was performed to detect variants that are significantly associated with this effect, as reflected by a reduction in UPDRS Part III total score. On average, poor responders did not exhibit any improvement in the score after 12 weeks of zonisamide treatment, whereas responders achieved a mean decrease of 9.9 (*P* = .73 × 10^−8^) (Supplementary Table 3). The two patient groups had similar demographic and clinical characteristics except for baseline UPDRS Part III total score, which was higher in responders than in poor responders (23.6 ± 10.0 vs 15.7 ± 10.6, *P* = 0.0113).

None of the SNPs or genes investigated in the GWAS showed a genome-wide significant association with a reduction in UPDRS Part III total score (Supplementary Figs. 11–13). The four SNPs showing a suggestive association (*P* < 1 × 10^−5^) with this parameter are listed in Supplementary Table 4. However, in the subsequent pathway-based analysis, six canonical pathways and 12 GO terms (e.g., transmission across chemical synapses, axonogenesis, and neurogenesis) showed a genome-wide significant association (Supplementary Table 5). In addition, processes associated with calcium signaling also showed a suggestive association with improvement in the motor symptoms of PD patients.

## Discussion

To our knowledge, this is the first GWAS to investigate the genetic basis of the response to an antiparkinsonian drug. SNP-, gene-, and pathway-based association analyses revealed that chromosome 1q32.1 and several pathways were significantly associated with a reduction in “off” time by zonisamide treatment in PD patients. This association was independent of the baseline “off” time value and the daily dose of zonisamide administered to patients.

The SNP (rs16854023) showing the strongest association with a reduction in “off” time in the GWAS is located between the *MDM4* and *LRRN2* genes. More than 60% of patients with the TT genotype of this SNP showed a reduction in “off” time of over 1.5 h, and responsive TT carriers showed a greater than sevenfold decrease in mean “off” time compared to carriers of alternative genotypes (1.42-h vs 0.19-h). Evidence from the eQTL data suggests that several SNPs on 1q32.1 that showed a suggestive association in the GWAS influenced *MDM4* expression in tibial nerve tissue. For instance, homozygous responsive allele (C) carriers at SNP rs10900597 showed an over 24-fold decrease in “off” time compared to homozygous carriers of the alternative allele (T) in our study (Supplementary Fig. 8); and according to eQTL data from GTEx, subjects with the CC genotype had higher expression of *MDM4* in the tibial nerve (Supplementary Fig. 7a).

On the other hand, the lack of association between these SNPs and other clinical variables (Supplementary Fig. 9a, b) implies that the association between these SNPs and the reduction in “off” time are not influenced by the severity of PD and “wearing-off”. In addition, these polymorphisms do not appear to alter zonisamide metabolism; in fact, our data indicate that the reduction in “off” time is independent of the zonisamide dosage. Interestingly, the lack of association between these SNPs and changes in the UPDRS part III total score of PD patients suggests that zonisamide may act via different mechanisms to improve motor symptoms of PD patients. This finding is supported by results from the GWAS of UPDRS Part III total score (Supplementary Tables 4 and 5) that revealed sets of SNPs and pathways distinct from those identified in the GWAS of “off” time. Due to the characteristic of our study subjects who were typical “wearing-off” patients with good “on” levels (i.e., who responded well to levodopa and other standard drugs) but experienced a shorter drug effect [7], we were unable to identify variants significantly associated with improvement in motor symptoms, as reflected by a reduction in UPDRS Part III total score.

The *MDM4* gene encodes MDMX, an ubiquitin ligase that is a negative regulator of the tumor suppressor gene p53 [27]. Although the role of MDMX in PD is unclear, it is known to be critical for neuronal survival [28, 29], which may be related to its inhibition of p53. While increased p53 activity in PD promoted neuronal apoptosis [30, 31], dopaminergic neuron-specific deletion and pharmacological inhibition of p53 reduced the loss of dopaminergic neuron [31] and motor deficits [32, 33], respectively. In our study, carriers of genotypes associated with higher *MDM4* expression (GTEx portal) responded more favorably to zonisamide treatment and achieved a greater reduction in “off” time. Since motor fluctuations in advanced PD may result from the progressive loss of dopaminergic presynaptic terminals, which decreases dopamine storage capacity [34], our finding supports a potential neuroprotective effect of zonisamide in reducing dopaminergic neuron loss through a p53-mediated mechanism, although the details thereof require clarification through functional studies.

The results of our pathway-based analysis are in accordance with those of previous studies and suggest that calcium-, glutamate-, and potassium-related pathways mediate the antiparkinsonian effects of zonisamide [35–39], although other novel pathways may also be involved. For instance, GTPases including Ras GTPase identified in the GWAS of “off” time have been previously reported to play a role in PD pathogenesis and are potential therapeutic targets [40]. Similarly, pathways associated with axonogenesis and neurogenesis identified in the GWAS of UPDRS strongly support a neuroprotective role for zonisamide. However, validation of our findings in independent subjects and follow-up functional analysis of the *MDM4* gene are necessary to translate our findings into better management of “wearing-off” in PD. The latter would be facilitated in clinical settings by determining of patient genotype and predicting their response to zonisamide in order to identify those who are most likely to benefit from this drug treatment.

Most pharmacogenetic studies on the response to antiparkinsonian drugs have been limited to investigations of candidate genes [41, 42]. Ours is the first hypothesis-free study examining genes on a genome-wide scale. Additional studies of this nature should provide more clues for clinicians to identify patients who are more likely to respond well to a drug so that the most appropriate intervention can be prescribed. This would lead to better management of “wearing-off” in PD through precision treatment.

## Supplementary information

Supplementary Figures

Supplementary Table 1

Supplementary Table 2

Supplementary Table 3

Supplementary Table 4

Supplementary Table 5
