# Supplementary material for: Genome-wide association study identifies zonisamide responsive gene in Parkinson’s disease patients
Source: J Hum Genet. 2020 May 1;65(8):693–704. doi: 10.1038/s10038-020-0760-8 (PMC8075945; doi:10.1038/s10038-020-0760-8)
Supplement: Supplementary file 1 — Supplementary Figures [file 10038_2020_760_MOESM1_ESM.pptx]

## Slide 1
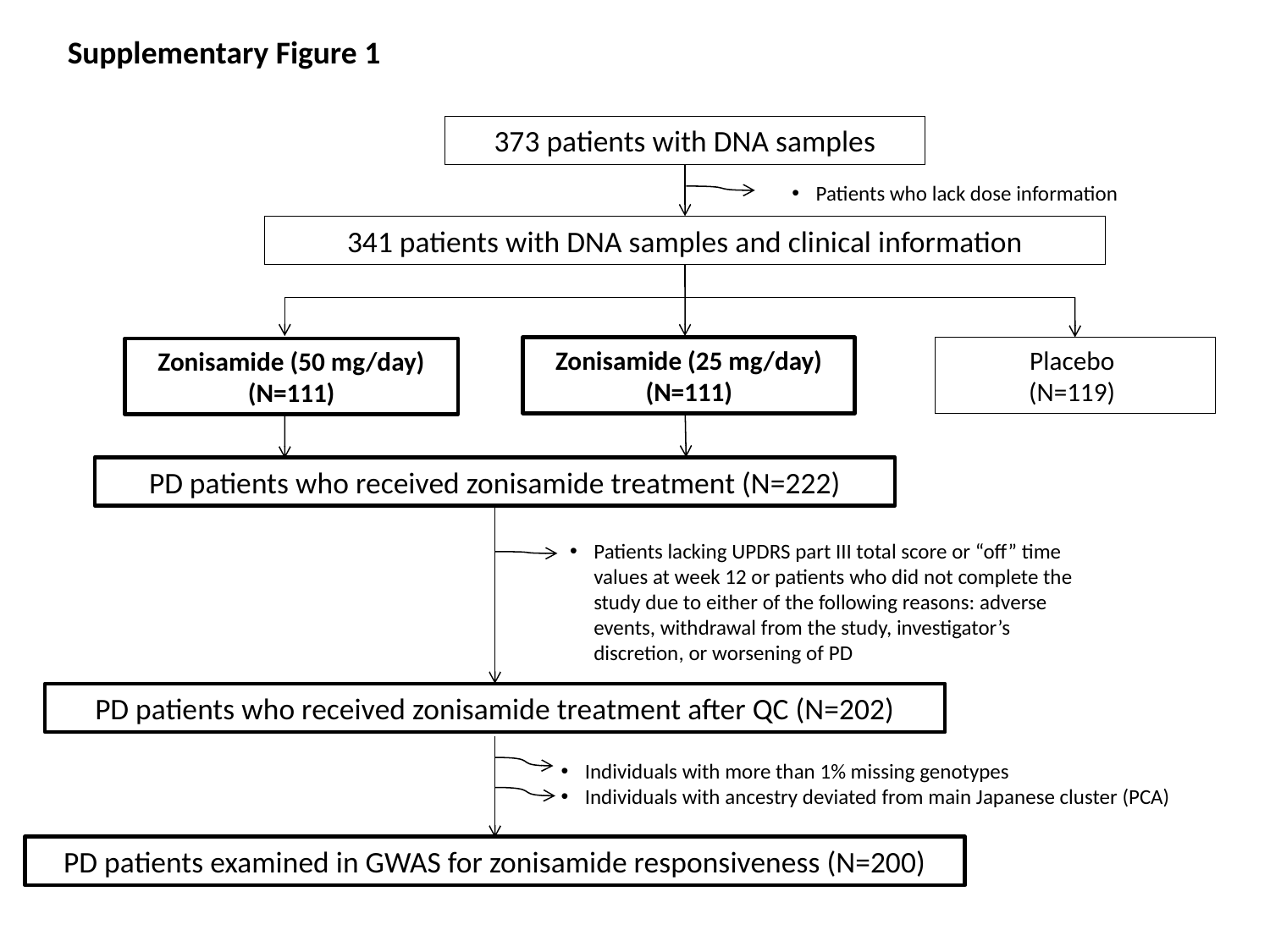

Supplementary Figure 1
373 patients with DNA samples
Patients who lack dose information
341 patients with DNA samples and clinical information
Zonisamide (25 mg/day)
(N=111)
Placebo
(N=119)
Zonisamide (50 mg/day)
(N=111)
PD patients who received zonisamide treatment (N=222)
Patients lacking UPDRS part III total score or “off” time values at week 12 or patients who did not complete the study due to either of the following reasons: adverse events, withdrawal from the study, investigator’s discretion, or worsening of PD
PD patients who received zonisamide treatment after QC (N=202)
Individuals with more than 1% missing genotypes
Individuals with ancestry deviated from main Japanese cluster (PCA)
PD patients examined in GWAS for zonisamide responsiveness (N=200)

## Slide 2
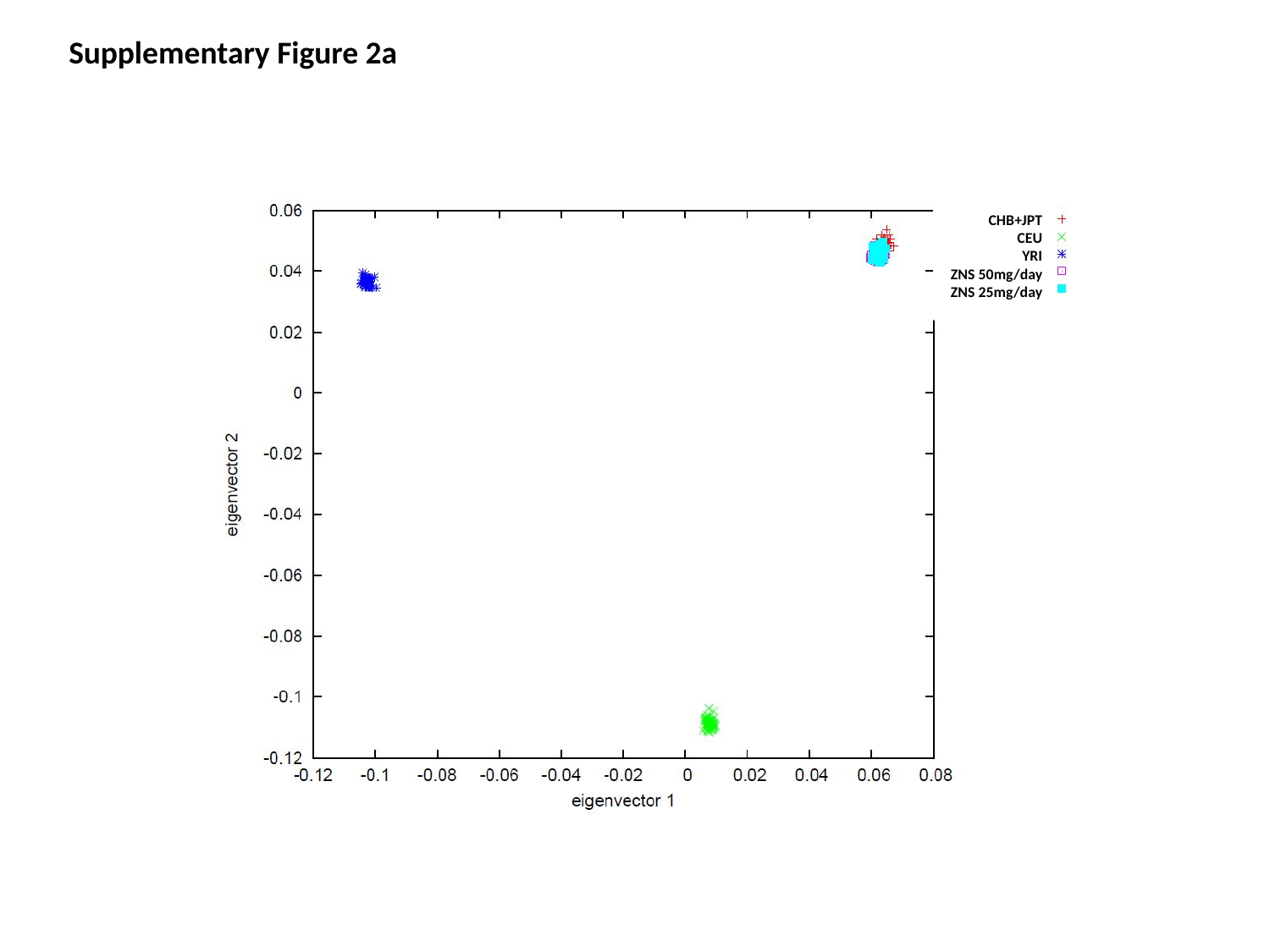

Supplementary Figure 2a
CHB+JPT
CEU
YRI
ZNS 50mg/day
ZNS 25mg/day

## Slide 3
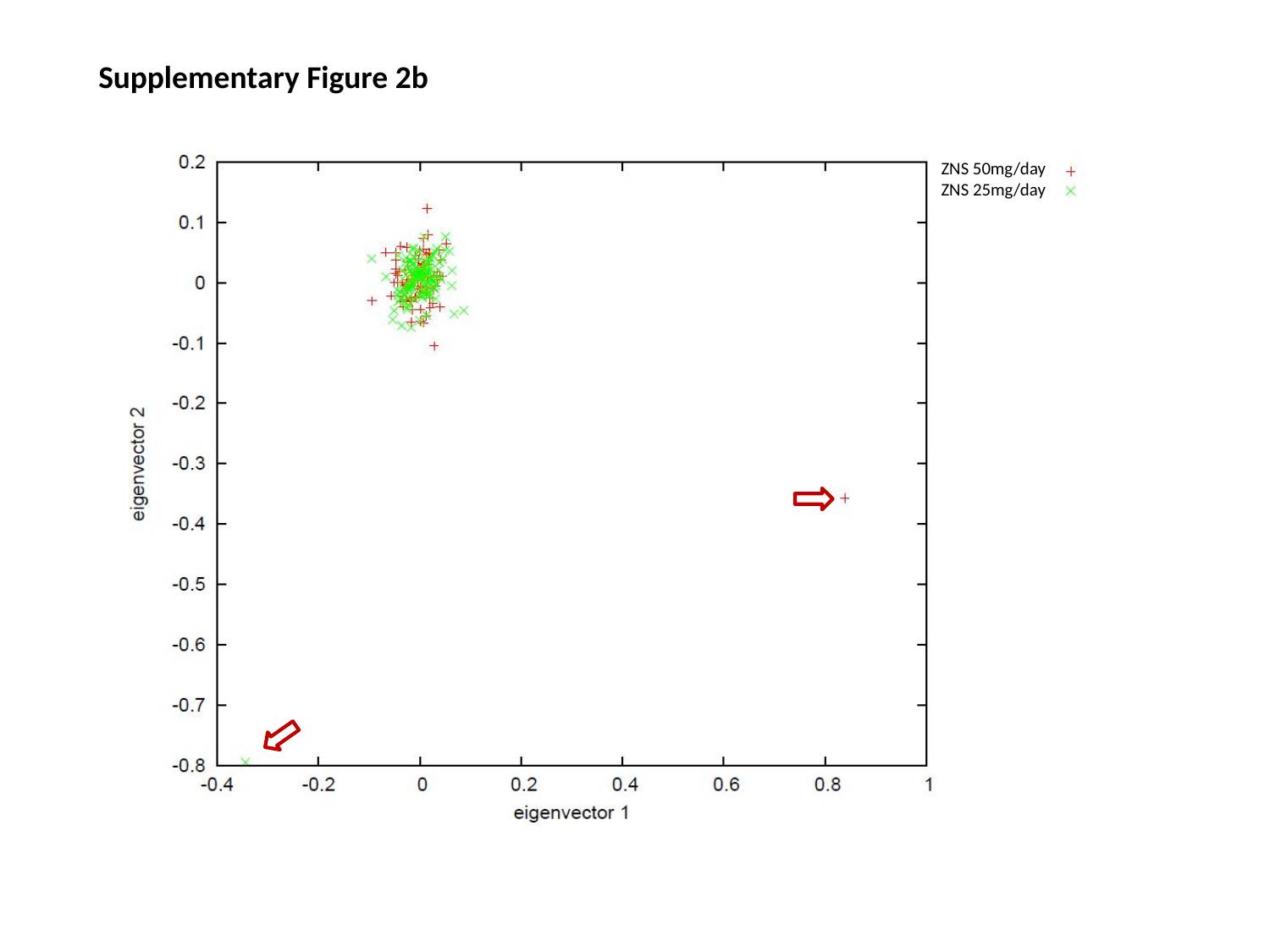

Supplementary Figure 2b
ZNS 50mg/day
ZNS 25mg/day

## Slide 4
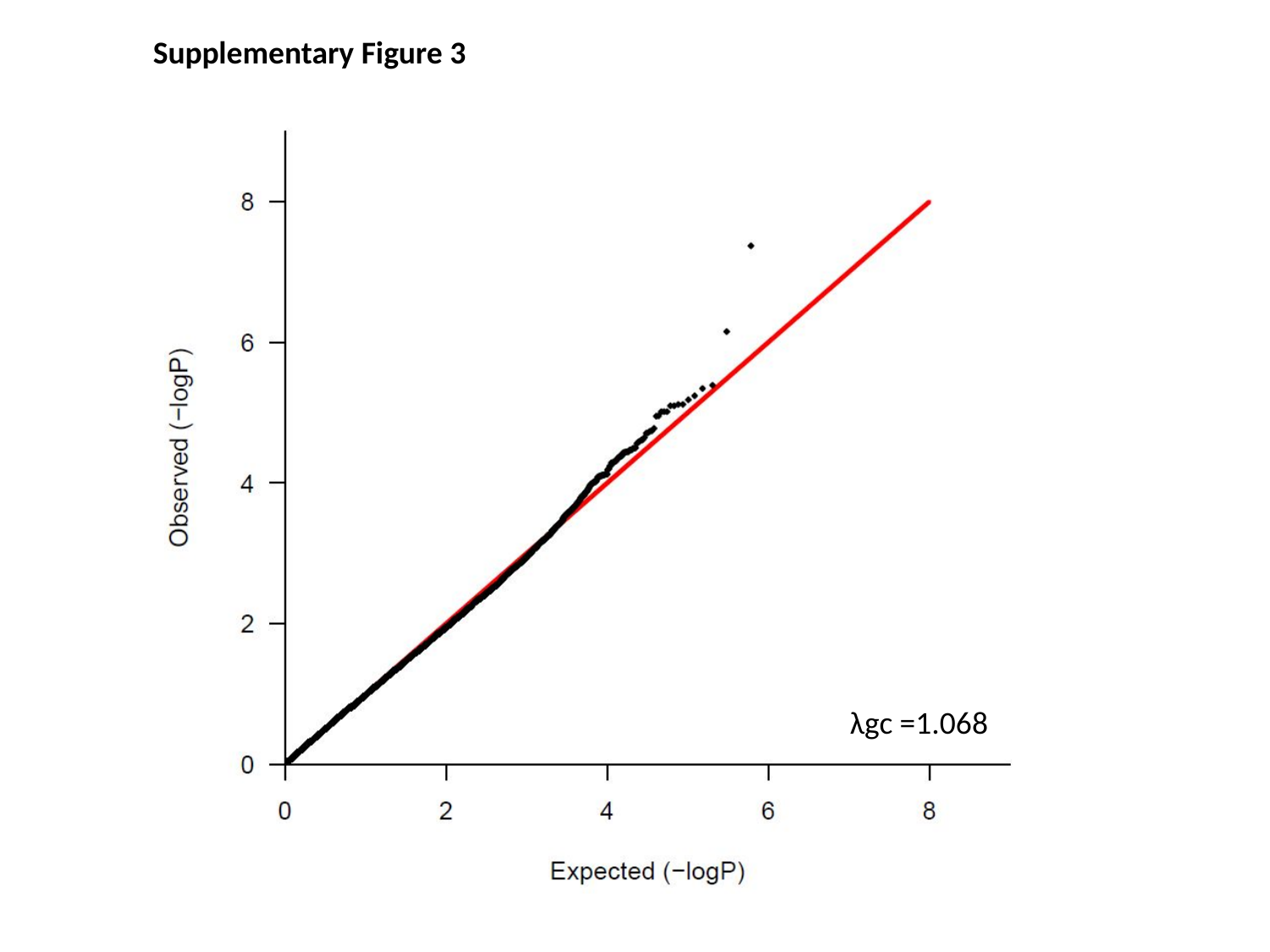

Supplementary Figure 3
 λgc =1.068

## Slide 5
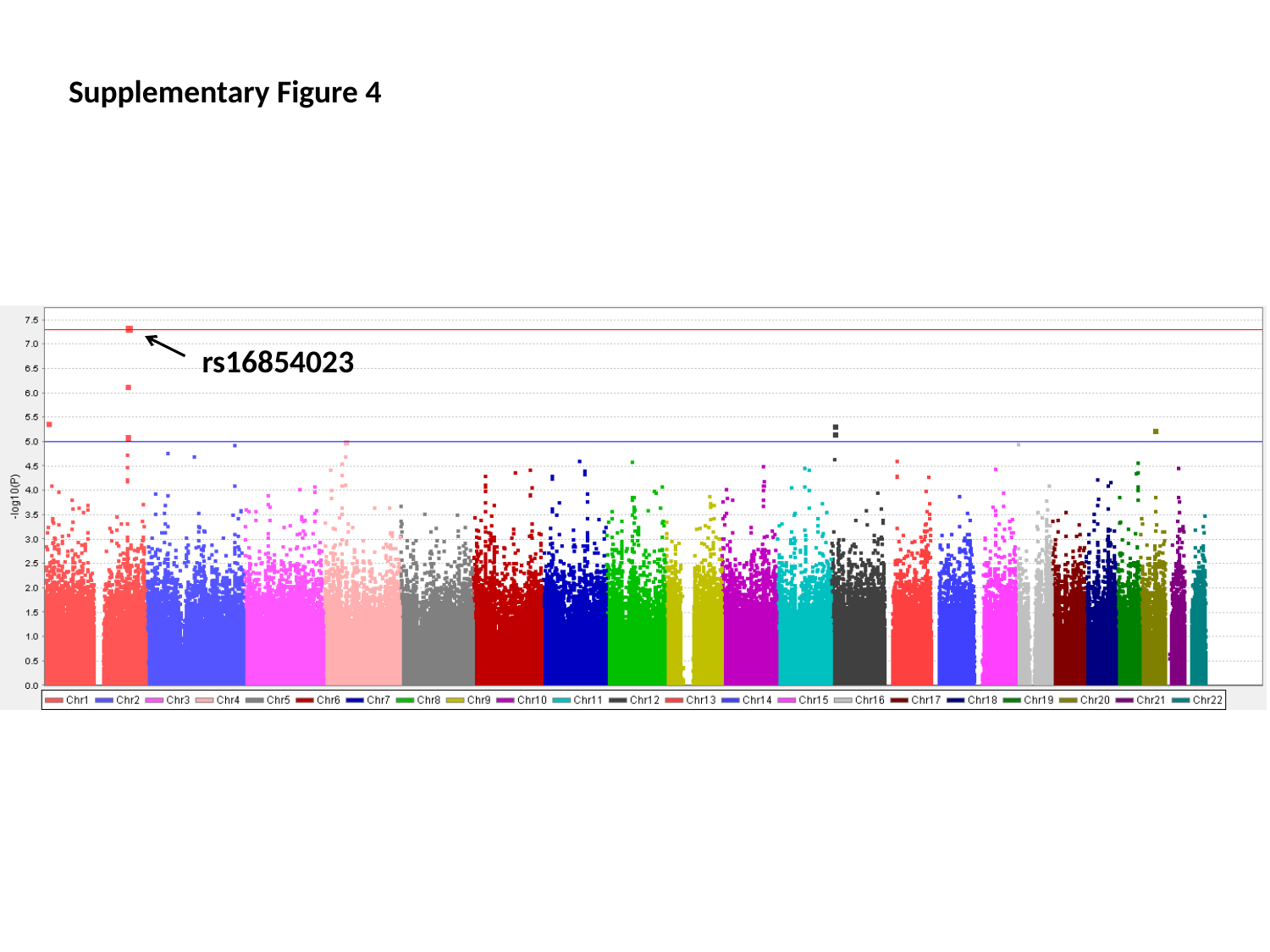

Supplementary Figure 4
 rs16854023

## Slide 6
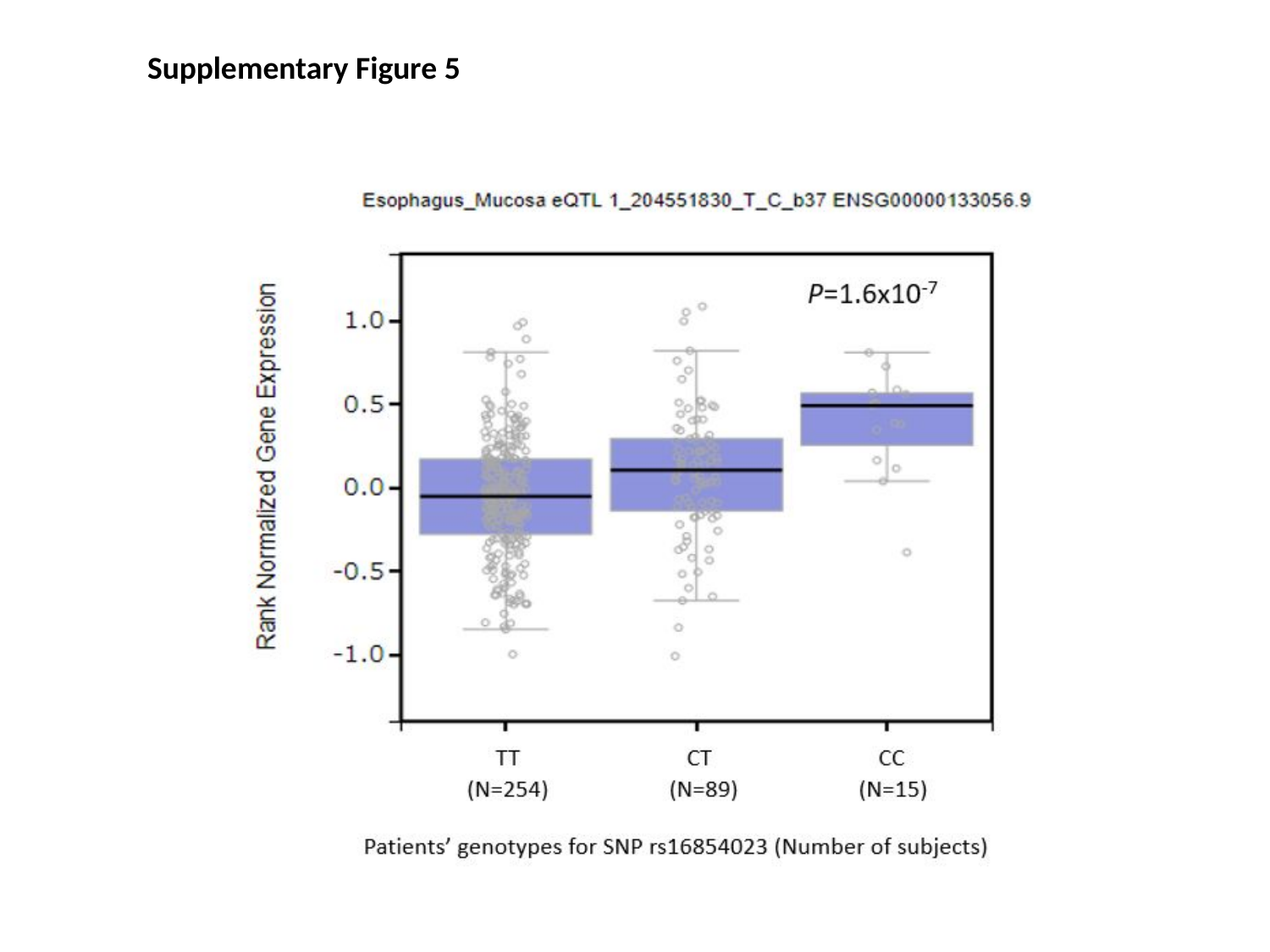

Supplementary Figure 5

## Slide 7
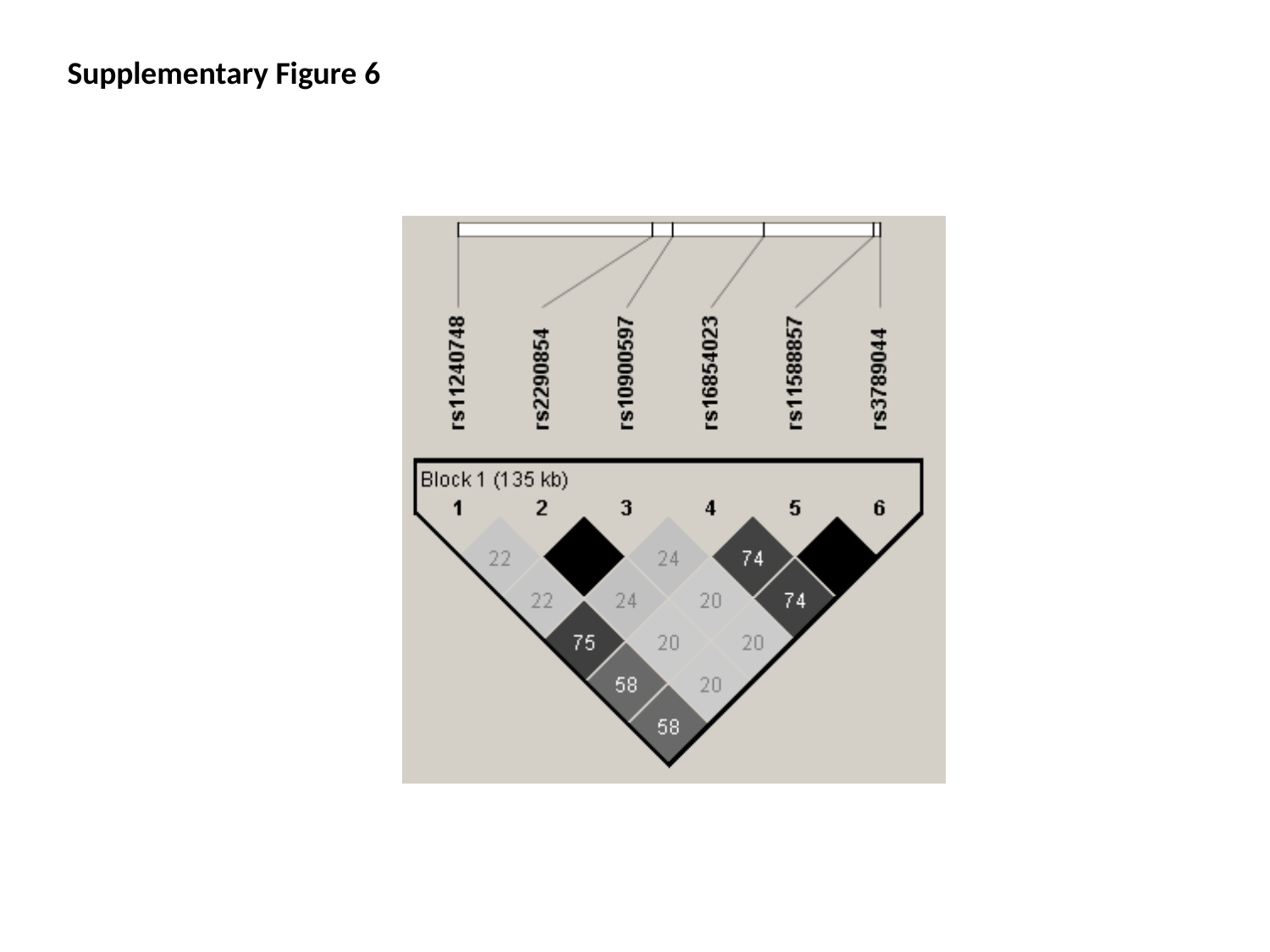

Supplementary Figure 6

## Slide 8
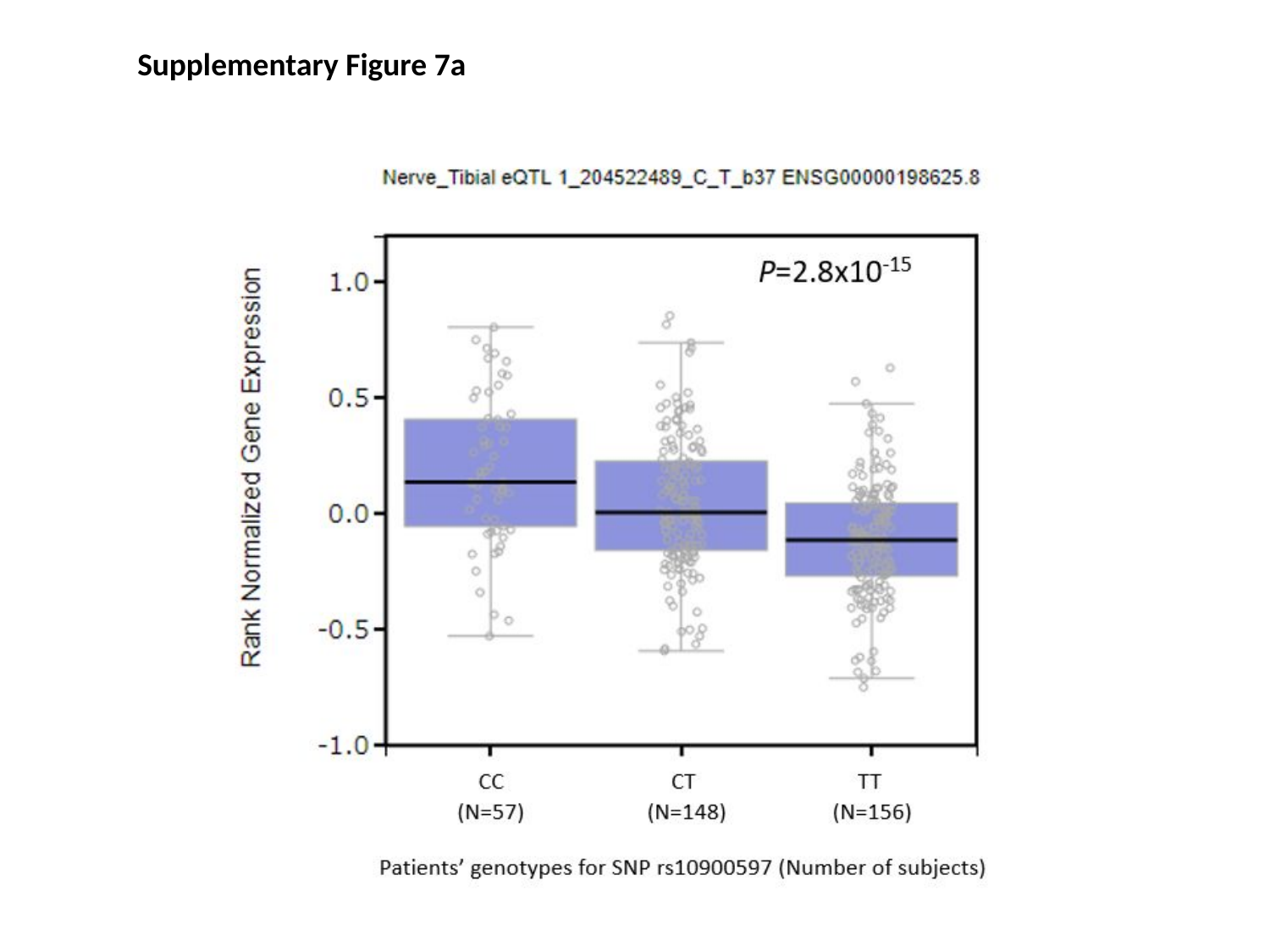

Supplementary Figure 7a

## Slide 9
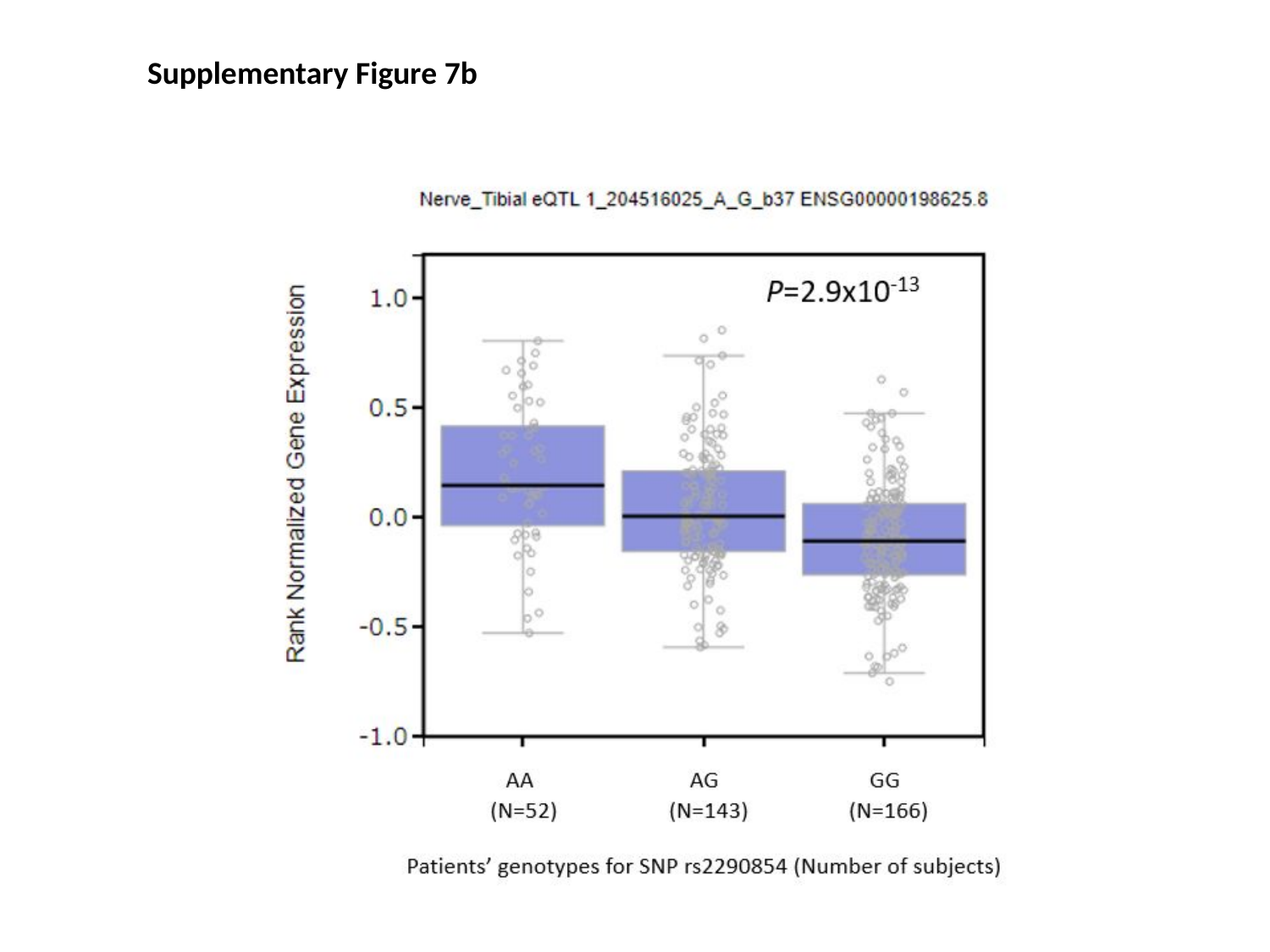

Supplementary Figure 7b

## Slide 10
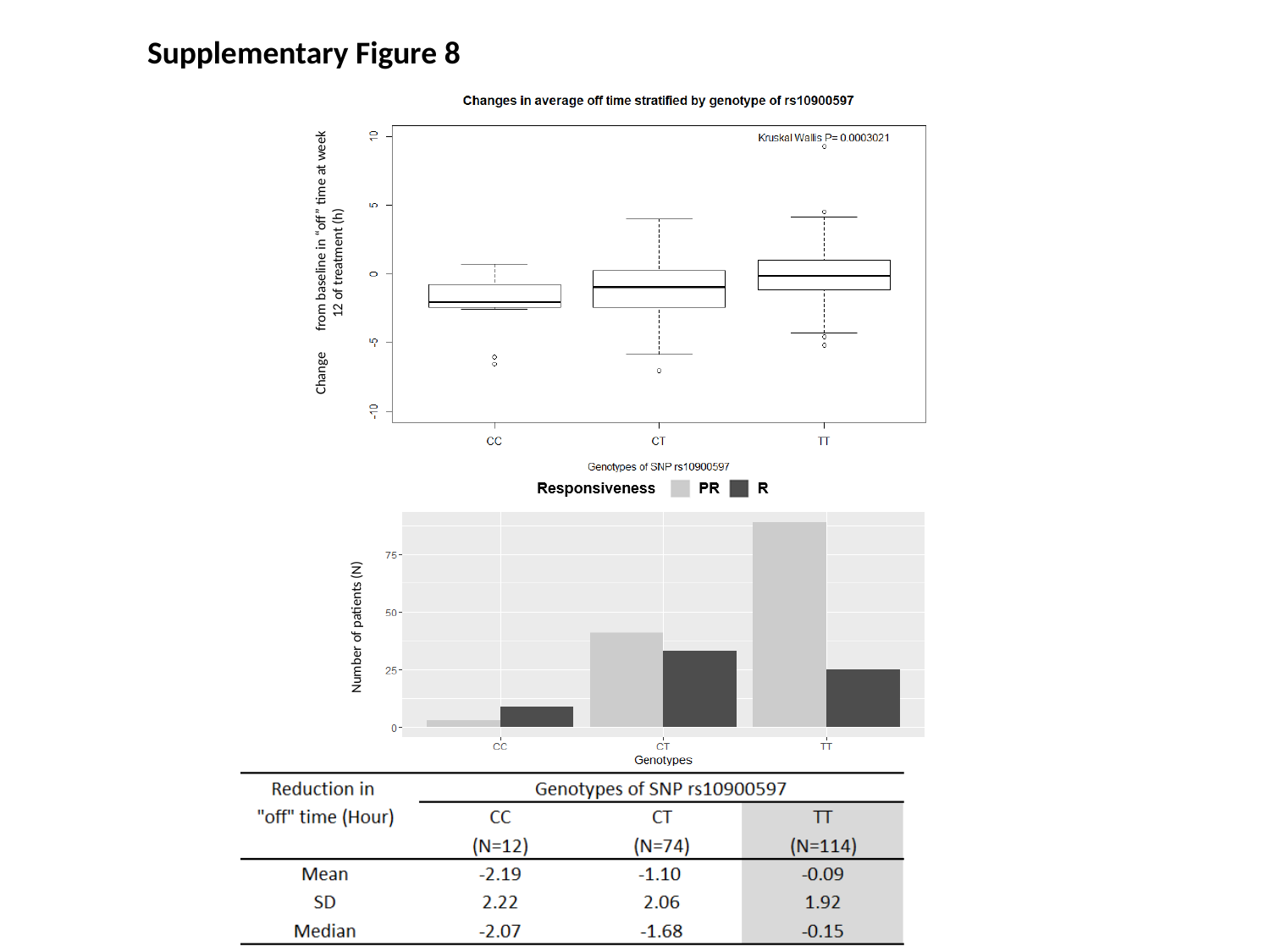

Supplementary Figure 8
Change　from baseline in “off” time at week
12 of treatment (h)
Number of patients (N)

## Slide 11
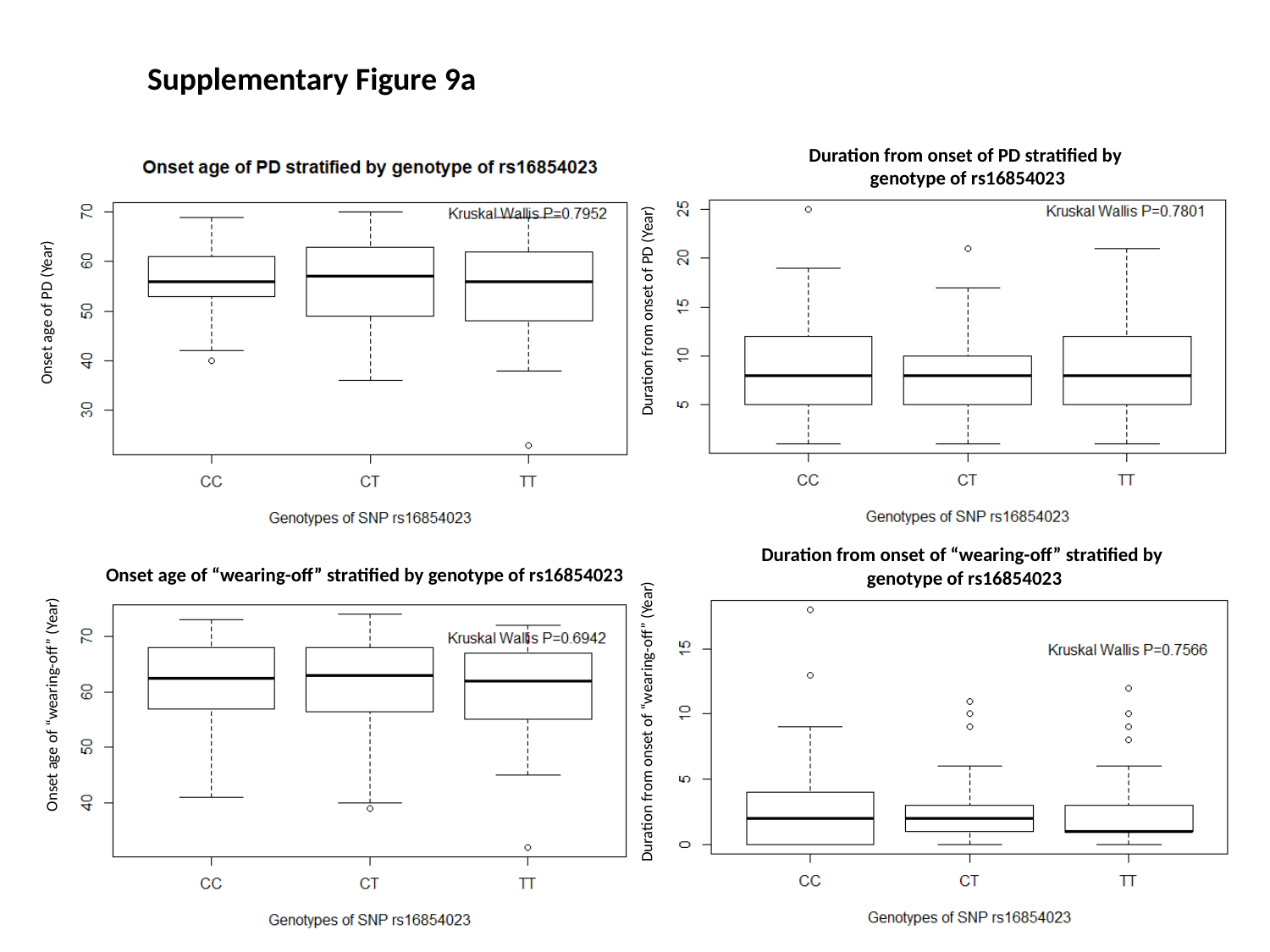

Supplementary Figure 9a
Duration from onset of PD stratified by
genotype of rs16854023
Duration from onset of PD (Year)
Onset age of PD (Year)
Duration from onset of “wearing-off” stratified by
genotype of rs16854023
Onset age of “wearing-off” stratified by genotype of rs16854023
Onset age of “wearing-off” (Year)
Duration from onset of “wearing-off” (Year)

## Slide 12
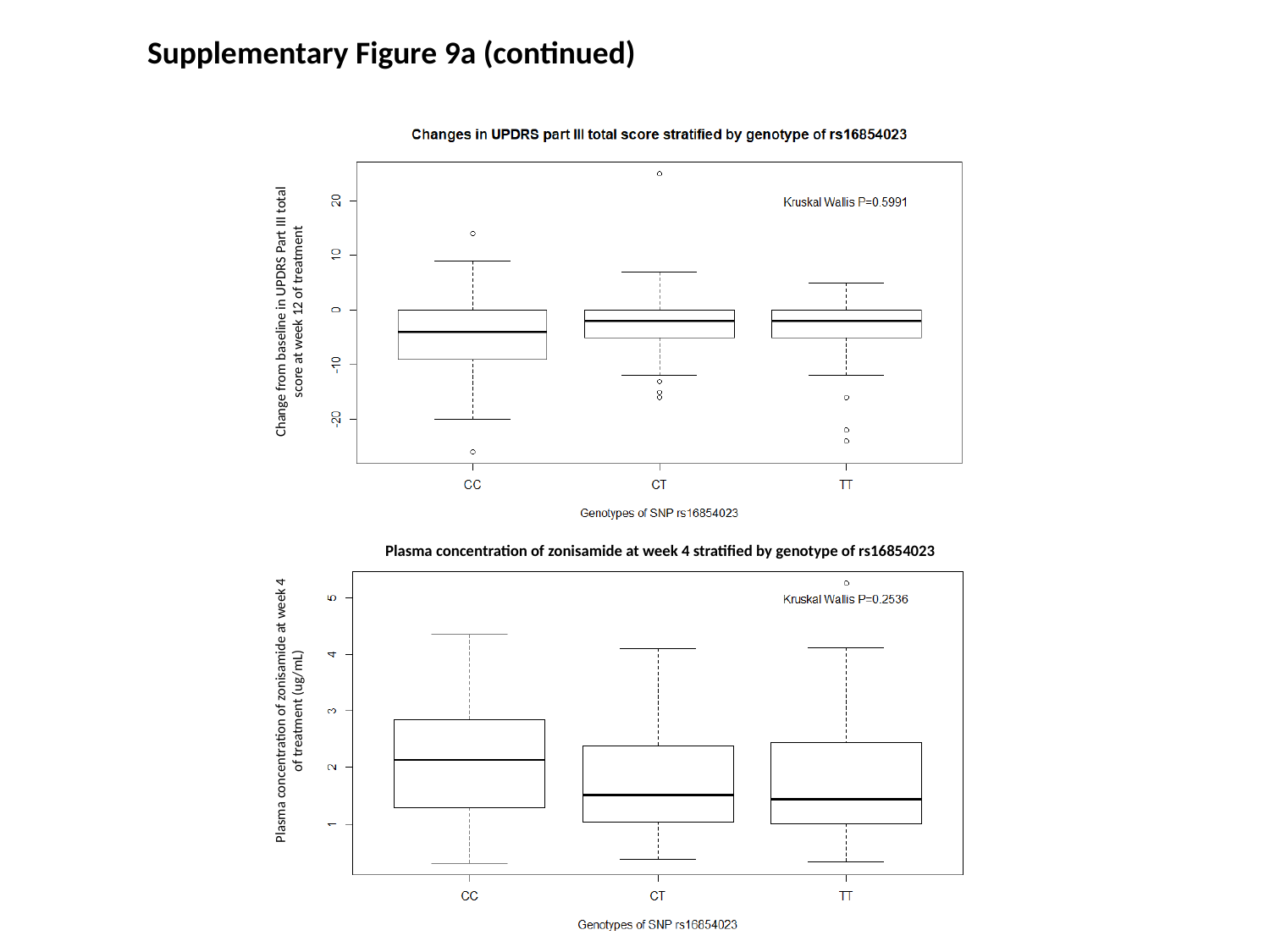

Supplementary Figure 9a (continued)
Change from baseline in UPDRS Part III total score at week 12 of treatment
Plasma concentration of zonisamide at week 4 stratified by genotype of rs16854023
Plasma concentration of zonisamide at week 4 of treatment (ug/mL)

## Slide 13
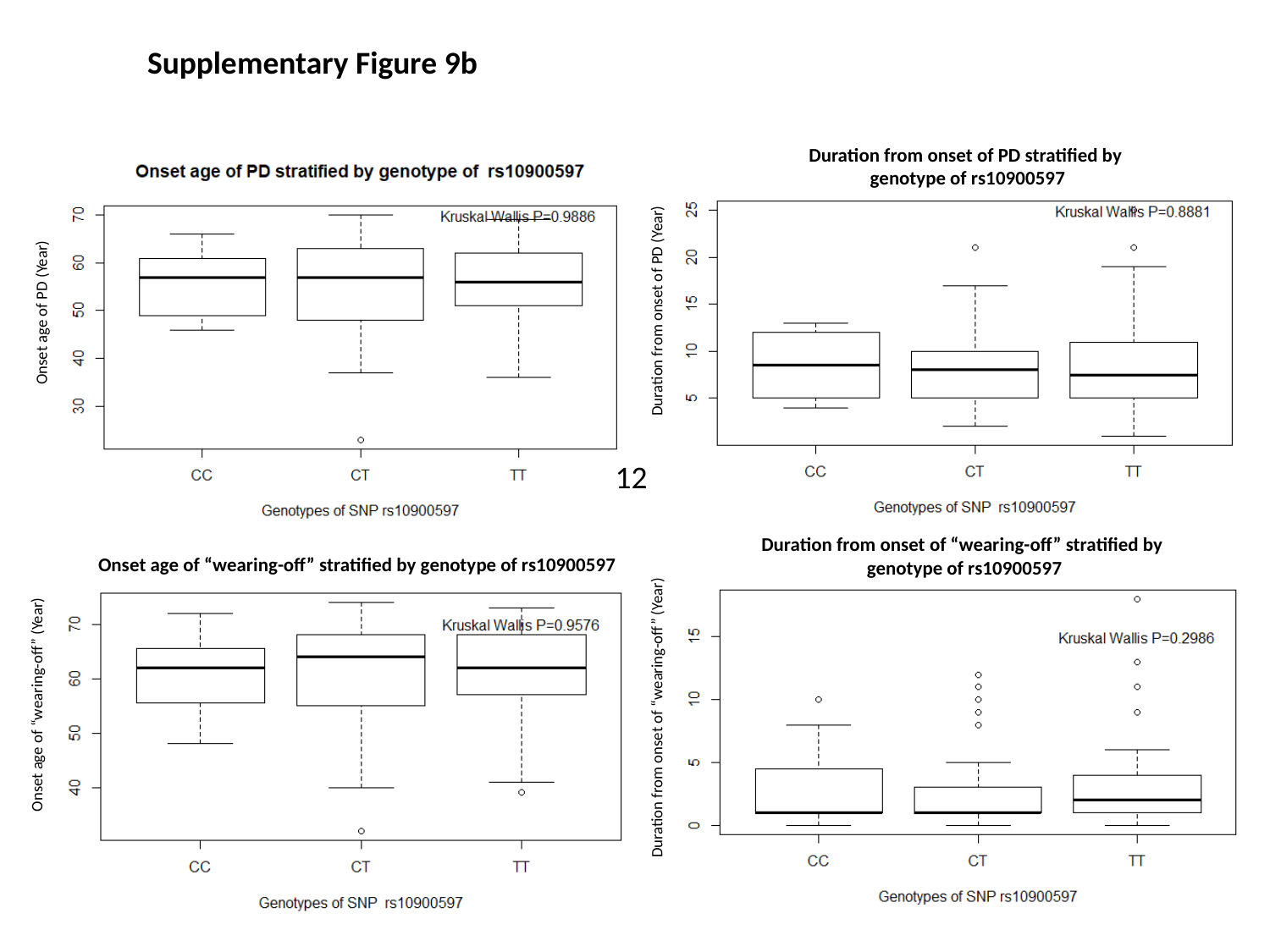

Supplementary Figure 9b
Duration from onset of PD stratified by
genotype of rs10900597
Duration from onset of PD (Year)
Onset age of PD (Year)
12
Duration from onset of “wearing-off” stratified by
genotype of rs10900597
Onset age of “wearing-off” stratified by genotype of rs10900597
Onset age of “wearing-off” (Year)
Duration from onset of “wearing-off” (Year)

## Slide 14
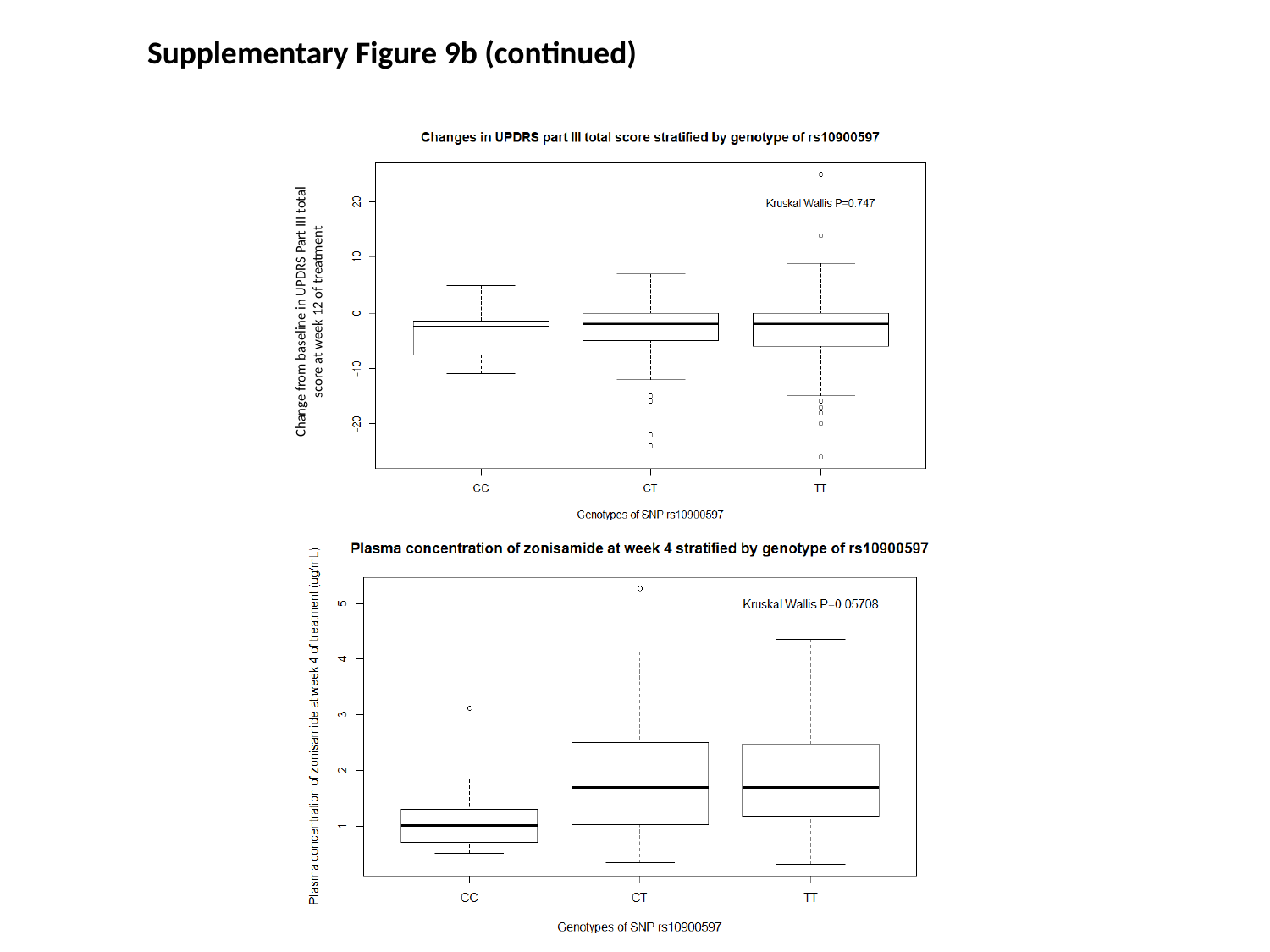

Supplementary Figure 9b (continued)
Change from baseline in UPDRS Part III total score at week 12 of treatment

## Slide 15
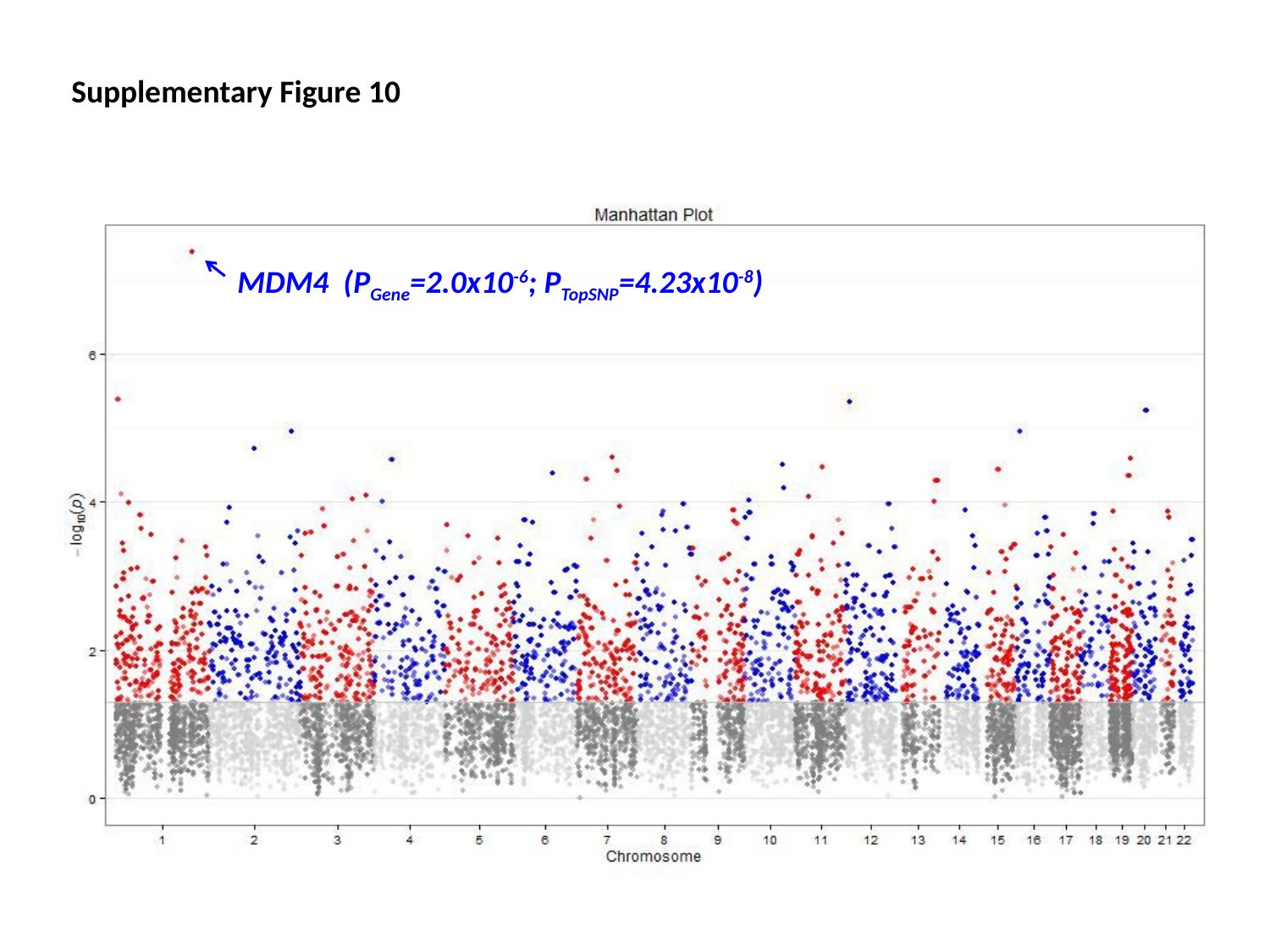

Supplementary Figure 10
MDM4 (PGene=2.0x10^-6^; PTopSNP=4.23x10^-8^)

## Slide 16
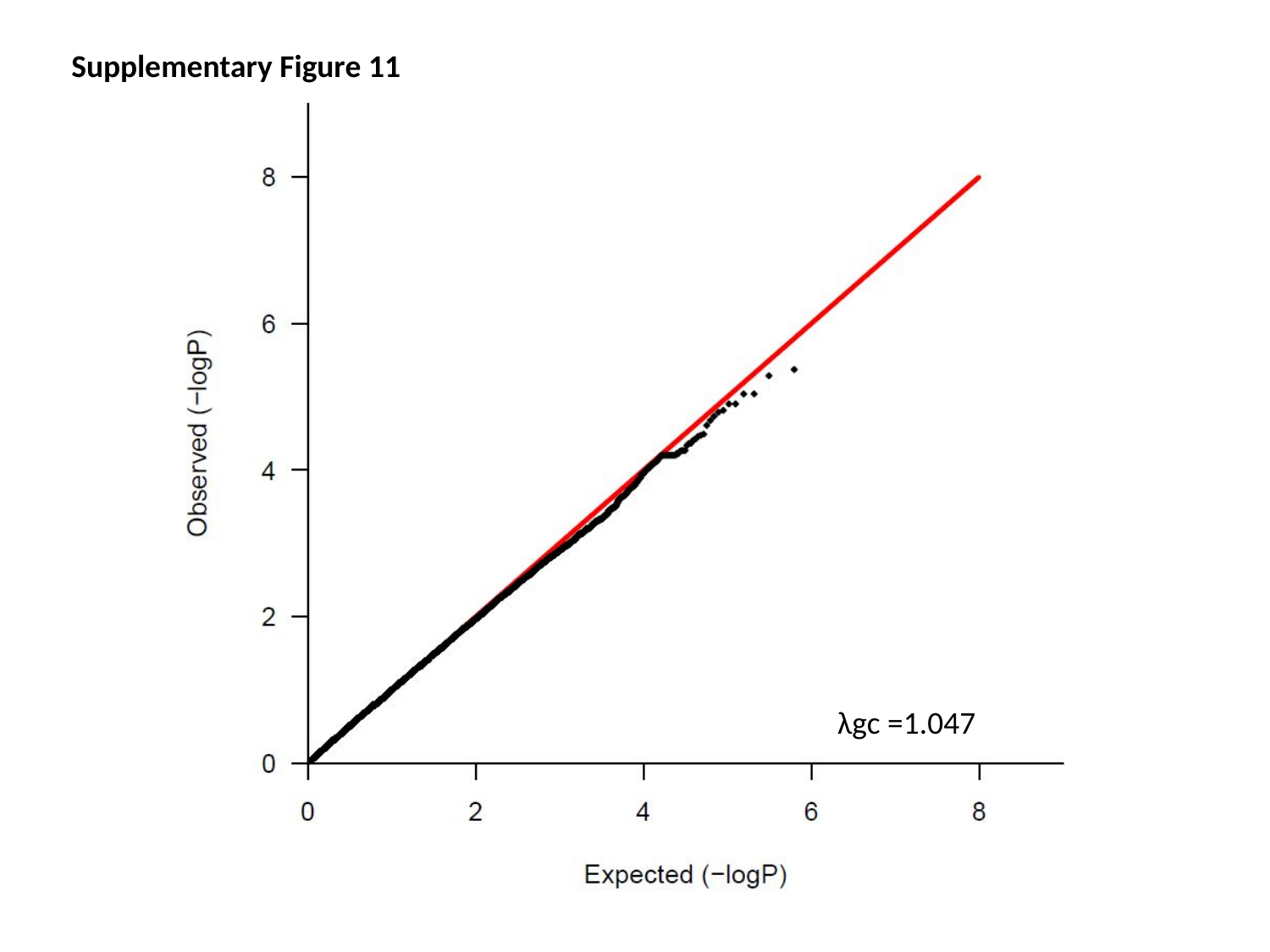

Supplementary Figure 11
λgc =1.047

## Slide 17
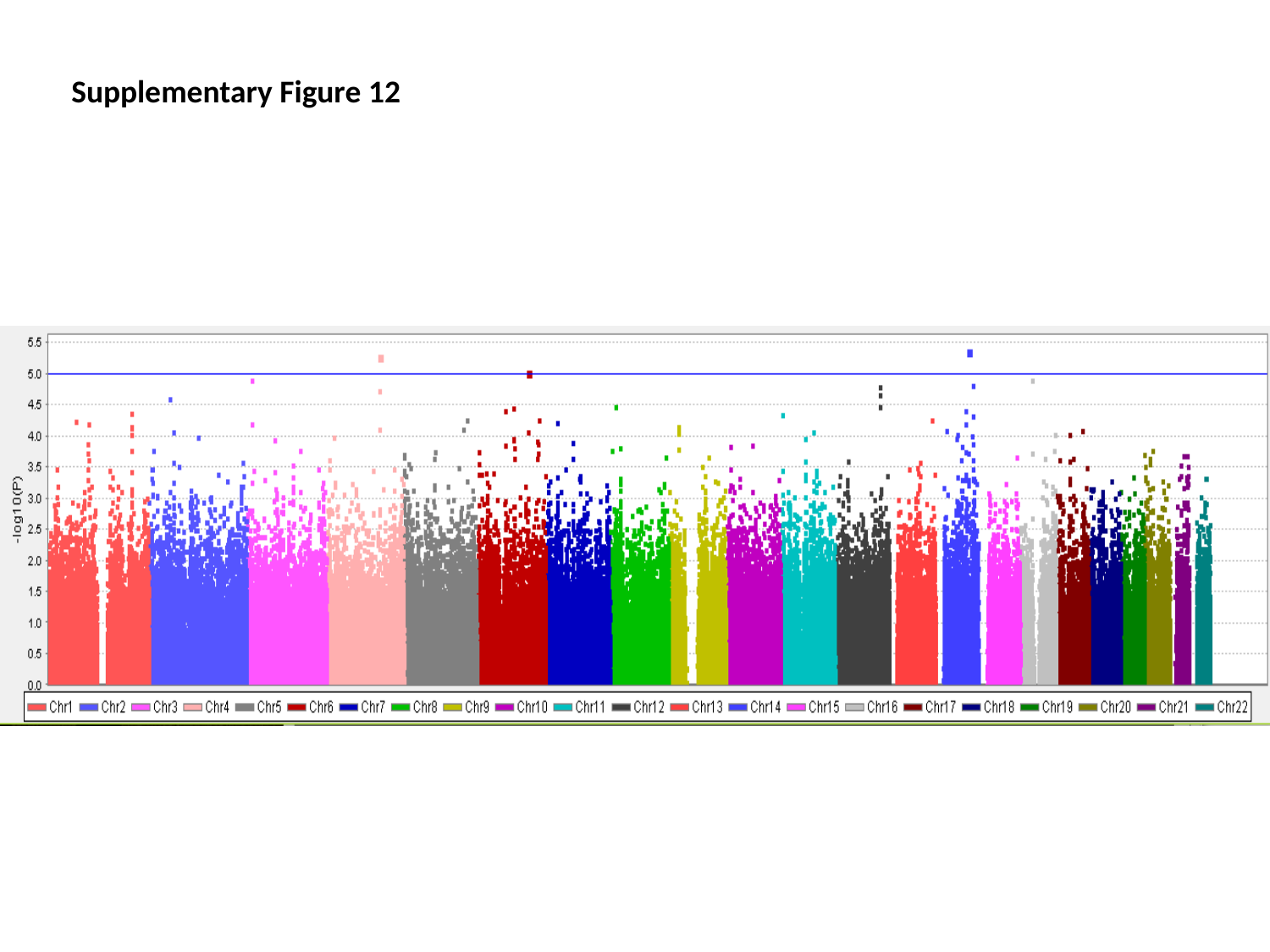

Supplementary Figure 12

## Slide 18
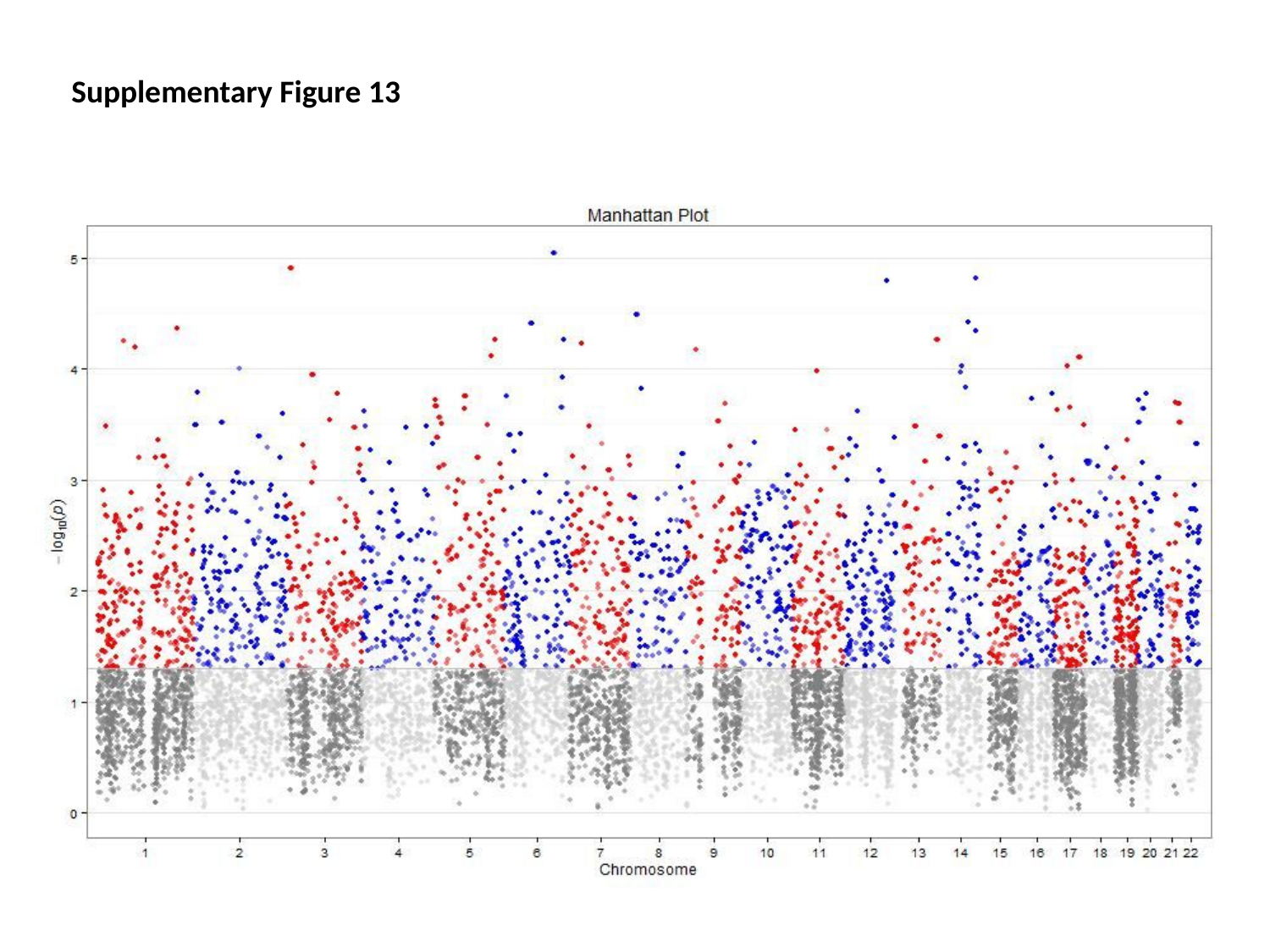

Supplementary Figure 13
